# Needs Assessment for the Development of an Electronic Cross-Facility Health Record (ECHR) for Pediatric Palliative Care: A Design Thinking Approach

**DOI:** 10.3390/children8070602

**Published:** 2021-07-16

**Authors:** Theresa Sophie Busse, Chantal Jux, Sven Kernebeck, Larissa Alice Dreier, Dorothee Meyer, Daniel Zenz, Boris Zernikow, Jan Peter Ehlers

**Affiliations:** 1 Department of Didactics and Educational Research in Health Science, Faculty of Health, School of Medicine, Witten/Herdecke University, 58448 Witten, Germany; chantal.jux@uni-wh.de (C.J.); sven.kernebeck@uni-wh.de (S.K.); jan.ehlers@uni-wh.de (J.P.E.); 2 PedScience Research Institute, 45711 Datteln, Germany; l.dreier@pedscience.de (L.A.D.); d.meyer@pedscience.de (D.M.); b.zernikow@kinderklinik-datteln.de (B.Z.); 3 Department of Children’s Pain Therapy and Pediatric Palliative Care, Faculty of Health, Witten/Herdecke University, 58448 Witten, Germany; 4 Smart-Q Softwaresystems GmbH, 44801 Bochum, Germany; zenz@smart-q.de; 5 Pediatric Palliative Care Centre, Children’s and Adolescents’ Hospital, 45711 Datteln, Germany

**Keywords:** digital health, health information technology, innovation, process model, user-centered design, palliative care, palliative medicine, pediatrics, design thinking

## Abstract

Background: Pediatric palliative care (PPC) is characterized by years of multisectoral and multi-professional care. Sharing information between PPC professionals is, therefore, essential for quality care. The evidence shows that electronic cross-facility health records (ECHRs) provide useful support in this context. To our knowledge, no ECHRs have been developed through a user-centered approach for this specific setting in Germany. Methods: Guided by design thinking, first, qualitative interviews were conducted to assess the needs of PPC professionals. Second, the elicited needs were specified in focus groups (FGs). Based on the needs stated in the interviews, prototypes of the ECHR were developed and discussed in the FGs. The indicated needs were supplemented and specified in an iterative process. The prototypes were further adapted according to these results. The unified theory of acceptance and use of technology was the basic model in the evaluation of needs. Results: Across seven main categories, past and current medication, emergency view, and messaging functions were identified as the participants’ desired core components of an ECHR. Utilizing design thinking facilitated the explicit articulation of user needs. Conclusions: Developing an ECHR with the content identified would allow for real-time data during emergencies, tracking what other PPC professionals have done, and making the applied treatments visible to others. This would offer a broader picture of the complex conditions common to PPC.

## 1. Introduction

Pediatric palliative care (PPC) represents a holistic patient and family centered care approach for neonates, children, adolescents, and young adults with life-limiting or life-threatening illnesses—that is preferably provided by a multi-professional team (e.g., physicians, nurses, psychosocial staff, and therapists) [1,2]. PPC patients’ diseases are often rare and characterized by a high variance of symptomatology [3,4,5]. Furthermore, due to frequent fluctuations in the patient’s health status, rotating between inpatient and outpatient settings is common.

In Germany, care during the relatively stable phases of an illness is provided at home, supported by general practitioners and pediatricians as well as general outpatient pediatric palliative care teams. If necessary, specialist doctors or specialized outpatient pediatric palliative care (SOPPC) teams are provided. When symptoms worsen, inpatient admission to pediatric palliative care units (PPCUs) occurs. Outpatient hospice services and hospices offer soothing relief regardless of the severity of symptoms and provide support to the family (e.g., with a stay of up to four weeks annually in a children’s hospice). Outpatient hospice services can relieve families by taking over everyday activities and offering discussion opportunities on the topics of grief, death, and bereavement. Hospices allow patients to be admitted for respite care as well as space for self-help [6].

In Germany, there are currently two PPCUs as well as some extra designated places in hospitals for PPC. In addition, there are at least 15 children’s hospices. Care by SOPPC teams is almost nationwide in Germany [1].

Regarding the collaboration between the various professions in different sectors, clear and comprehensible documentation is vital, yet this poses a special challenge [7]. To our knowledge, the documentation in the various facilities is currently still analog or in non-specific electronic documentation systems (e.g., systems for adult palliative care or systems for basic pediatrics). The information collected and documented in one setting must be displayed as clearly and quickly as possible to providers in other settings. This applies to the entire period of care and can, therefore, be necessary over an extended period. Electronic cross-facility health records (ECHRs) may constitute a viable solution to this [8]. An ECHR facilitates the exchange of patient-related data between PPC professionals in different settings, lists a patient’s medical history, and may contain patient information such as diagnoses, medication, treatment plans, information on allergies, and diagnostics [8]. To the best of our knowledge, no ECHR for PPC exists in Germany.

A system tailored to the needs of users, daily practice, and healthcare delivery structures is essential to an innovative and practical ECHR in PPC. To adapt the technology to professionals’ needs and ensure user acceptance, those users should be involved in its participatory design [9]. One approach frequently employed during participatory design is design thinking (DT) [10]. DT is a method characterized by multidisciplinary, creative collaboration [11] in an iterative process that consists of multiple steps and aims to fully understand a specific problem to develop a comprehensible, effective solution [10].

User acceptance is a known issue with implementing a new electronic health record, such as an ECHR. It plays a key role in the system’s actual use after implementation and user satisfaction with it [12]. Individually and collectively, the expected usefulness, technical concerns, technical problems, and expected workflow challenges are conducive or obstructive to the acceptance of technologies [13]. The unified theory of acceptance and use of technology (UTAUT) is often used to examine the acceptance of technologies. The UTAUT focuses on various certain determinants that influence whether technologies are accepted and perceived as useful by users, such as performance expectancy, effort expectancy, social influence, and facilitating conditions. The UTAUT defines performance expectancy as the function that users consider useful. Effort expectancy primarily refers to the effort and complexity of using new technology. This also includes usability aspects. In addition, age, gender, experience, and voluntariness affect intention. Social influence may be, for example, the positive coaxing of friends or superiors, while some facilitating conditions are, for example, high internet bandwidth or the enabling of regular training sessions [14].

Given the lack of ECHR for PPC, this study aimed to integrate DT to capture how an ECHR might support, facilitate, and meet the specific needs of inpatient and outpatient PPC professionals (physicians, nurses, and secretaries). In addition, it sought to develop an example ECHR as a mock-up based on these needs. The study rests on the following underlying research question:

What are the needs of potential future users in the setting of PPC with regard to the development of an ECHR?

## 2. Methods

This study is part of the ELSA-PP (Electronic Intersectoral Record System for Palliative Care) project. Ethical approval was obtained from the Ethics Committee of Witten/Herdecke University, Germany (approval code: 35/2019).

### 2.1. Theoretical Framework

An adapted six-step DT model was used to analyze the current status of the intersectoral PPC exchange and PPC professionals’ ECHR needs (needs assessment; steps 1–3), and, based on this, to conceptualize an appropriate ECHR (conceptualization; steps 4–6; Table 1).

While DT made up the basic methodology, the UTAUT model is a great decision for the content design and analysis of the needs. The use of the model should help to investigate which factors are of great relevance for the ECHR and with which additional arrangements the acceptance of the technology can be increased. The UTAUT model’s broad focus [14] should enable a perspective that goes beyond the sole consideration of content or usability. This is critical when implementing an ECHR that provides a completely new approach to information sharing in order to gain a real advantage over traditional methods in PPC.

### 2.2. Sampling

PPC professionals (nurses, physicians, and secretaries) from medical offices, SOPPC teams, and a PPCU were asked to participate in the study.

A total of *n* = 222 physicians from medical offices (pediatricians and general practitioners) were contacted; first by mail, then by e-mail or telephone. All of them had experience with PPC patients.

A total of 25 nurses, 2 physicians, and 1 person from administrative staff of the PPCU were invited to participate in the study. The participating PPCU was the first of its kind to open in Europe in 2010, bringing many years of PPC experience to its multi-professional team. Some people were also employed at the local SOPPC team in addition to their work at the PPCU. These five nurses, three physicians, and one person from administrative staff were also asked to participate in the study and share their impressions from both the PPCU perspective and the SOPPC team perspective. Contact was established by mail and via a notice on the bulletin board of the PPCU as well as personal contact.

Additionally, the employees of SOPPC teams (twelve nurses, eight physicians) were invited to participate in the study. Here, again, the first contact was made by mail, and then by e-mail or telephone.

All persons who were contacted received detailed verbal and written study information as well as a demographic questionnaire, providing their written informed consent prior to participation. During every step of the study, participants received an expense allowance of EUR 40 per hour.

### 2.3. Data Collection and Procedure

**Figure 1:**
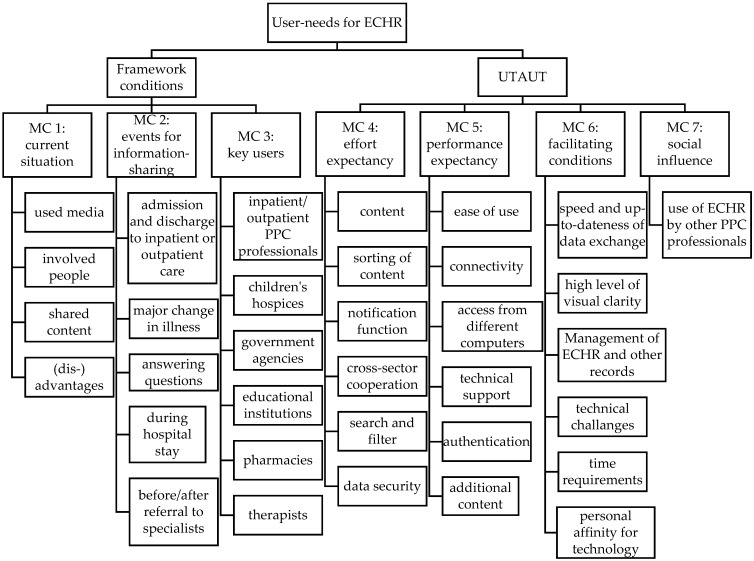
Extract from the category system.

Semi-structured interviews [16] with PPC professionals were conducted as the basis for the needs assessment (Step 1—Empathy). Their findings offer insights into the current state of information sharing and professionals’ ideas as to future information exchange. The interviews allowed (potential) users to report their relevant needs, desires, and experiences (Figure 1) for ECHR development.

The interviews were conducted by telephone or video call due to the participants’ heavy workload and to account for the need for contact reduction in the context of the COVID-19 pandemic. All interview guidelines were previously created through discussions and consensus finding in the research and development team.

During the next step of data collection, PPC professionals were contacted again and invited to participate in FGs (Step 5—Test). Participation in the interviews was not a mandatory requirement for this step. The FGs integrated DT by initially raising awareness among participants about the need for a PPC ECHR. Visual tools (a patient journey map, stakeholder map, and mock-ups) were used to present content in a comprehensible and appealing way and to increase creativity [16]. A patient journey map (Figure S1, [17]) was presented using a fictitious patient to emphasize the frequent sector changes of PPC patients. A stakeholder map (Figure S2) of all those providers and facilities identified in step 1 with which the participants currently exchange patient data was exhibited. The FGs were then asked to identify which of the providers and facilities should have (a) read access, (b) write access, or (c) no access to the ECHR. So-called mock-ups were then presented to the participants based on the professionals’ needs analyses gleaned from interviews. The mock-ups functioned as click prototypes of the ECHR user interface that included the required content, a rough basic structure, and the various desired functions. The FGs were conducted as videoconferences and their audio was recorded using an external recording device for subsequent transcription. The FGs were also accompanied by artists performing graphic recording [18] (Figure S3). After completion, the graphical records were reviewed with the participants to ensure that all key information was mapped and critical discussion points identified.

Following the FGs, the research team revised and adjusted the mock-ups’ design and usability to meet practitioners’ needs, and then compiled the final conceptualized ECHR (Step 6—Iteration).

### 2.4. Data Analysis

The interview recordings were transcribed verbatim according to the transcription rules of Dresing and Pehl [19] (Step 2—Define). The transcripts were subsequently evaluated via content analysis according to Kuckartz’s concept, using MAXQDA software [20]. The UTAUT was considered in the formation of the categories.

The analysis was initially performed by two independent researchers (T.B. and C.J.), who then compared their results in a continuous exchange and created the code system. Finally, the codes were reviewed and discussed by the study’s other researchers (S.K., D.M., L.D.) until a consensus was reached.

A key element from the agile software development methodology Scrum was applied in this step [21]. The professionals’ needs identified in the interviews were translated through the Scrum method into narrative “user stories” in a standardized manner (e.g., “*As a user, I would like to be able to view a child’s current emergency medication to alleviate acute discomfort*”).

The research and development team then jointly discussed how to best meet the professionals’ needs (Step 3—Ideate). Through brainstorming sessions and team workshops, the ECHR’s possible design was structured based on the user stories.

After this, the mock-ups of the ECHR elements were developed by the research and development team using Balsamiq Wireframes software [22] (Step 4—Prototype) to prepare the FGs (Step 5—Test).

The FGs were analyzed according to the same principle as the interviews (Step 6—Iteration). However, no new category system had to be developed; instead, the already established system was used and adapted inductively where necessary. Additionally, user stories were formulated in the iteration from the elicited needs, which served as the framework for customizing the mock-ups.

The participants’ original quotes were translated into English for this manuscript. All participants were assigned pseudonyms in the following structure: Focus Group (FG)/Interview (I) number, profession—setting.

## 3. Results

A total of *n* = 32 PPC professionals responded positively to the request and attended the interviews (Table 2): these were *n* = 7 PPCU professionals (*n* = 1 nurse, *n* = 4 physicians, *n* = 2 secretaries), *n* = 3 SOPPC professionals (*n* = 1 nurse, *n* = 2 physicians), and *n* = 18 physicians from the medical office (MO). A total of *n* = 4 professionals (*n* = 3 physicians, *n* = 1 nurse) who were employed at the PPCU also at times worked on the SOPPC team. Therefore, these individuals are listed in an extra category labeled PPCU/SOPPC to indicate that they brought expertise from both perspectives. A total of *n* = 11 participants had experience with electronic documentation software. The interviews lasted 12–72 min each (an average of 32.4 min).

The second data collection step included three FGs with a total of *n* = 13 participants (PPCU: *n* = 2 physicians; SOPPC: *n* = 3 nurses, *n* =2 physicians; PPCU/SOPPC: *n* = 2 physicians; MO: *n* = 4 physicians). The duration of the FGs was 65–82 min (an average of 73.75 min). After the three interviews were conducted, data saturation was reached. A total of *n* = 9 participants took part in both the interviews and the FGs.

The following presents the outcomes of the interviews and FGs in an integrative way to facilitate a clear presentation of the final results. The category system comprised 1401 codes in seven main categories (MC), with 61 subcategories and 76 further subcategories (Figure 1). Three of the main categories stemmed from the compiled interview guide, and four were oriented towards the UTAUT (correspondingly labeled in the following).

The corresponding interview guide-based categories were structured to focus on the ECHR’s needs and include the categories of *(MC 1) current situation, (MC 2) information sharing*, and (*MC 3*) *key users.* These categories were intended to clarify the framework conditions for the ECHR’s implementation. They, therefore, incorporate technical equipment in various settings as well as what times specific users need access and with what intention. The additional main categories were based on the UTAUT, including *(MC 4) effort expectancy*, *(MC 5) performance expectancy, (MC 6) facilitating conditions*, and *(MC 7) social influence*.

This chapter presents the results based on the category system, followed by a description of the developed mock-ups with their contents and functionalities in a comprehensive presentation of the results.

### 3.1. MC 1: Current Situation

This category mapped the current circumstances and content for patient data exchange. It identifies the advantages and disadvantages of the current information exchange to replicate the advantages in the ECHR and avert the continuation of disadvantages in the new system. The majority of participants reported using fax, telephone, or analogous documents (e.g., letters) to transmit patient data. The group of general practitioners/pediatricians reported using thirteen different electronic documenting systems during routine clinical practice. The participants reported unclear responsibilities, missing data, difficulty obtaining information, and various problems stemming from privacy regulations. Another frequently mentioned issue was the struggle to reach other professionals, which often requires several attempts, entailing that the exchange of information consumes a large amount of time.

*“When we now refer a child to the clinic in an emergency, then we know the explanation of the procedure in an emergency probably look at ourselves again, then print it out and then fax it there. But there are a lot of interfaces that do not work well, especially on a call. And when I have access to it, the fax may not yet have arrived in the emergency room but in the ward where the child has not yet arrived, eh? So, I think this targeted exchange of important information will remain with the telephone for the time being, but it would be so nice if you could already view the information in parallel* via *the digital path.”*

(I_01, physician—SOPPC).

The participants reported a large volume of various information and documents that are already being shared. In addition to reports (e.g., hospital discharge letters, findings, treatment notes, medical history, and final reports), this includes administrative content (e.g., prescriptions, referrals, information on medical aids, master data, relatives’ contact data, and other healthcare professionals’ contact data) as well as medical and nursing content (e.g., vaccination status, allergies, ventilation parameters, medication plans, nursing history, nutrition plan, and information on emergency procedures). Diagnostic data (e.g., laboratory diagnostics, imaging, and diagnoses) form another category of named content that is exchanged. A great deal of PPC information is also exchanged regarding family work (e.g., conversation content, personal network information, and information regarding the current treatment and care goals), while treatment planning occurs across professions and sectors through information exchanges (e.g., discarded treatment approaches, recommendations, information regarding the current treatment goal, and topics for future treatment and care).

### 3.2. MC 2: Events for Information-Sharing

This category collected statements regarding the relevant events in information exchanges. A patient’s shift to another sector, acute changes in a patient’s health status, and clarifications of ambiguities were identified as the most important intersectoral interfaces.

*“Especially when there are direct queries about things that are unclear, when we have changed something: medications on the ward, for example. Then, the question comes directly, ‘Why did you do that?’ Or, ‘why so and so, and can we continue with it?’ This is then always quickly clarified, and I think it’s also good for the general practitioners because they have the procedure directly at hand.”*

(I_01, physician—PPCU).

### 3.3. MC 3: Key Users

This category summarized which PPC providers should have access to the ECHR and in which way. It became clear that the needs for read and write access differ extensively depending on the particular user and patient, while the respective wishes varied greatly between the different teams and sectors. The FGs’ consensus was, therefore, that eligibility should not be linked to a profession or setting but should be case-by-case, granted according to the patient and their provider network.

*“That one considers who needs which information. This can also be different in each case; that is, it may very well be important to involve certain providers more or to provide them with certain basic medical information. Or, for example, to pass on certain things to the MDK [Medical Service of the Health Insurance Funds]. But also, to release very specific, so per individual, documents in principle.”*

(FG_01, physician—medical office).

### 3.4. MC 4—UTAUT: Effort Expectancy

The UTAUT defines effort expectation as a category that includes statements about the extent to which users believe that using the ECHR will help them achieve better job performance [14]. In the context of PPC, this included statements about how an ECHR could facilitate care routines and patient-centered information exchange.

The participants wished to be notified about new entries in the ECHR. This could be further specified in the FGs. For example, there was a desire to be notified by email when a new entry was made by others in an ECHR. However, these notifications ought to be individually configurable and thus, for example, not be sent for all patients or only for certain new entries. Simultaneous entries by different persons in the ECHR should be possible.

There was a desire for an upload function for files in different formats (JPEG, PDF, text documents, etc.). Support tools such as reminders, a to-do function, or the ability to create and edit checklists were mentioned as useful. It should also be possible for a user to enter their availability (e.g., “*calls preferably on Wednesdays between 12–14 o’clock*”).

Regarding the ECHR’s clarity, a keyword search with options to filter by creation date/type of document/posting person and a function to mark urgent or important information, to prioritize new content in the display, and to archive old content were emphasized. The period of the availability of documents should be adjustable and possible over a potentially long timeframe.

*“B: But if I now look at what it’s like with a, yes, what it’s actually like with a chronic patient, it’s too burdensome. It has to be ongoing. That’s, I think, also a problem that we have. Most ECHRs, as far as I know, yes, are created and only have a certain length of time, you know? And then it’s gone again. And that must not be with such a chronically ill or a palliative patient.”*

(I_05, physician—medical office).

A chronological overview of the most recent contacts and documentations, an emergency view where the most relevant patient data are available at a glance (emergency ventilation parameters, required medication, etc.), an overview of previous interventions in the form of a medical history, and the display of specific parameters (e.g., blood values) over a requested period were desired.

Within PPC, the ECHR should still be accessible after the child’s death, facilitating the organization’s follow-up care with the child’s relatives.

### 3.5. MC 5—UTAUT: Performance Expectancy

Performance expectancy in the UTAUT entails user expectation regarding the degree of simplicity of technology [14]. Accordingly, in our study, this category includes all the participants’ needs as to the ECHR’s ease of use.

The participants stated that they would like the ECHR to be simple to use with an interface that would allow them to transfer information from the ECHR to their documentation system.

*“Oh, which is perhaps another important thing; if this is an external program, then it should definitely have such a GDT interface so that you can integrate the data directly from this additional program into your software. That would perhaps be quite good?”*

(I_01, physician—medical office).

The participants identified certain content associated with this category that they would like to see added to the ECHR. In contrast to *MC 1*, this included content relevant for all professionals—from the perspective of the participants—that is not currently shared. The participants would like an overview of previous medical history, for example, in the form of a timeline where they can view all the previous hospitalizations, diagnoses, treatment attempts, therapies, and surgeries, sorted by time. The participants hoped this would provide a better understanding and overview of patients and their possible prospects for future treatment. They also wanted an emergency view with the most important information about a patient visible at a glance (e.g., diagnoses, ventilation, allergies, place of residence, advanced care plans, and symptoms). Advanced care plans, here, describe the documentation of wishes regarding resuscitation, medication administration in case of emergency, etc. In the specific setting, this is often recorded in the standardized document “declaration of emergency procedure”. Some patients also have an advance directive. The participants also valued an overview of every professional involved in a patient’s care. They stated that, currently, this is often unclear or unknown.

### 3.6. MC 6—UTAUT: Facilitating Conditions

The UTAUT defines facilitating conditions as the degree to which a person perceives an infrastructure that exists to organizationally or technically support the use of a system [14]. Accordingly, this category included factors that might at once limit and promote the participants’ willingness to use the ECHR.

To facilitate the use of the system, it should be accessible from various devices (smartphones, tablets, laptops, etc.), through, for example, a browser-based solution. Here, authentication should be as simple and secure as possible. Concrete ideas on this were mixed, ranging from SMS to e-mail tokens. Additionally, the desire for one-time access for people who only rarely need access to the ECHR was mentioned. Rapid and timely data exchange and digital availability were cited as encouraging factors to support the use of the system.

Participants feared technical problems and a large expenditure of time for using the ECHR; for instance, due to a lack of digital competence. Based on this, they identified the need for an ECHR that is clearly structured to provide easy navigation for less technically experienced users. In terms of the ECHR’s user infrastructure, there is, thus, a need for courses that prepare users to utilize the ECHR.

*“Well, in the end, of course, it’s always a bit of a problem that there are all kinds of, let’s say, affinities for technology. There aren’t? There are many who have a certain affinity and many who have no affinity at all […].”*

(I_09, physician—medical office).

### 3.7. MC 7—UTAUT: Social Influence

In the UTAUT, the factor of social influence states whether users think that significant others think that they should use the system [14]. In this category, the participants’ assumptions about how an ECHR might affect the social fabric were summarized.

The area of social influence was vaguely described by the interview participants. However, they mentioned that ECHR use would only make sense if all the PPC professionals involved in a child’s treatment use the same ECHR.

*“So, if this is, let’s say, open for extensions, then the usability factor would of course be much greater. So, if I can say that I can now communicate not only with my pediatric palliative team* via *such a thing, but perhaps also with my endocrinologist in the hospital outpatient clinic, or other things if the same system and the same functionality is behind it. Because if later the radiologists build their own system with us and the oncologists and whatever else, then of course it will be silly again.”*

(I_14, physician—medical office).

### 3.8. Final Design of the Conceptualized ECHR

The final design of the ECHR was developed in a mock-up based on the results from the needs assessment and as a final step of the conceptualization. The ECHR’s final design was developed in a mock-up that, in addition to the functions, also includes the various ways of viewing and exchanging content as well as editing methods.

In an ECHR suited to the PPC setting, the log-in process could involve a multi-factor authentication system that is suitable for different professions, does not require the installation of plugins or software, and meets privacy and security requirements.

**Figure 2:**
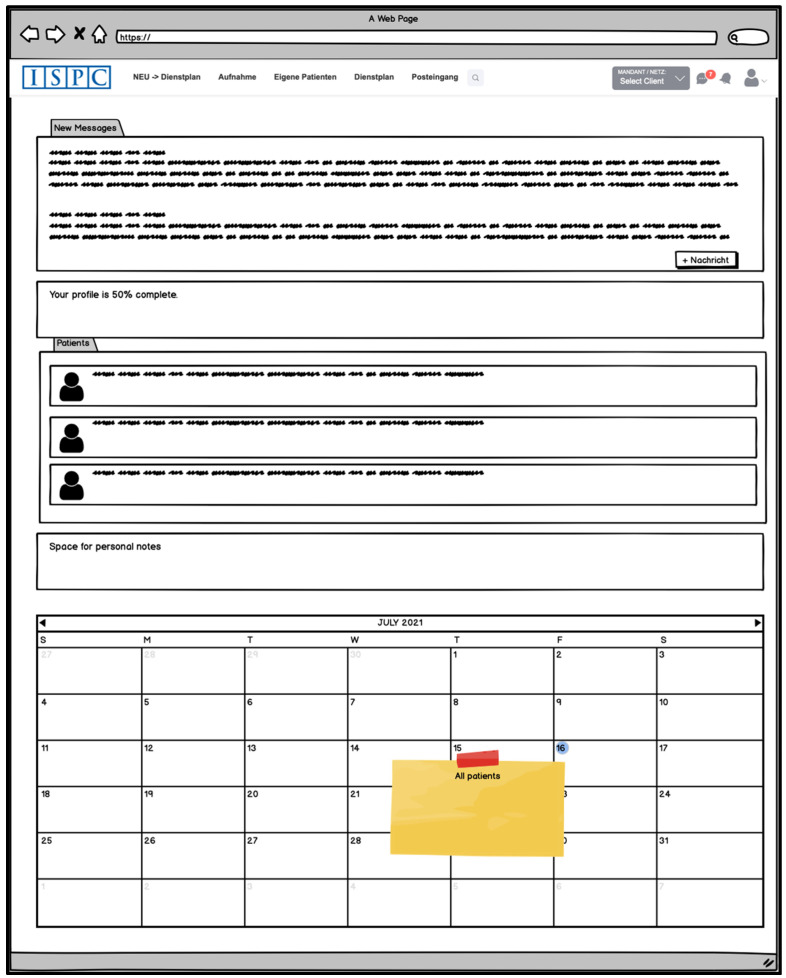
Idea for the superordinated starting page (part of the developed mock-up).

After logging in, users may be directed to a starting page (Dashboard) (Figure 2) that displays all the patients they care for. There might also be a text field for personal notes and a calendar displaying an overview of their patients’ appointments. The user could also view whether new ECHR entries have been made since their last login and if they have any new messages. Messages could be easily transferable between ECHR users via a corresponding function (“messenger”); for example, to arrange appointments regarding a patient’s treatment.

**Figure 3:**
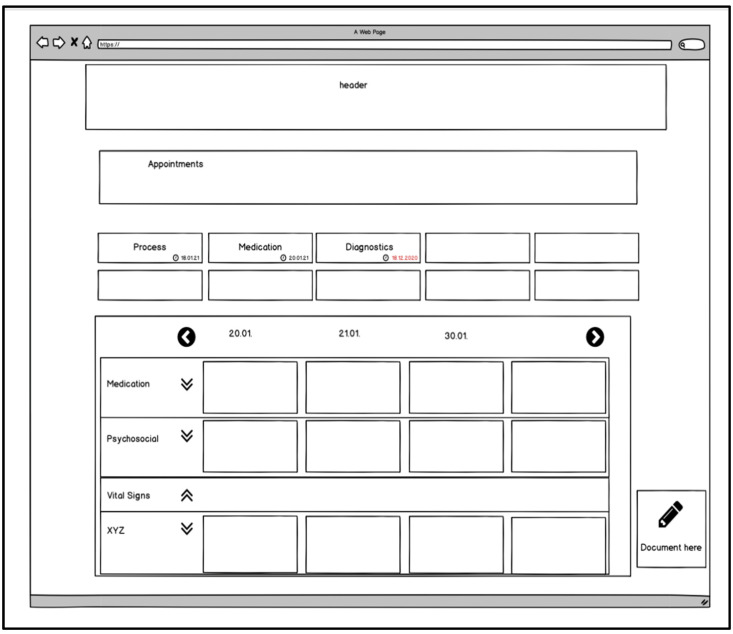
Idea for the patient main page (part of the developed mock-up).

If users select a patient on the superordinated starting page, they could be routed to the patient main page (Figure 3) where all the patient’s data and records are accessible. On this site, viewing and managing appointments or the “fever curve” could be displayed by default. In the “fever curve”, relevant medical care data such as medication, the documentation of vital signs, or treatment notes are displayed. In contrast to inpatient care, not every day of the year is displayed here, but only the days when documentation has been added to the “fever curve”.

Further subordinate sites and functions could be accessed from the patient main page. For instance, adjusting, uploading, and commenting on the following patient information could be possible: current medication, diagnoses, operations performed, current and past infections with multidrug-resistant pathogens, allergies, serious adverse events, and vaccination status. It might be helpful to list the possibility of documenting discarded therapies or therapies that were unsuccessful.

Users could also have the option of documenting the patient’s medical aids and which other persons or facilities are involved in the care process. Forms and applications could be integrated into the system via an upload function. Information on wounds, stomas, ventilation, and other special care features, such as ports or catheters, could be documented.

The much-desired medical history could reflect the often years’ long PPC by giving an overview of all of a patient’s diagnoses, inpatient stays, times of outpatient care, contacts by other PPC professionals, changes in therapy and operations, and special events (e.g., death of a caregiver, change in verbal skills, etc.).

There could be a “header” on all the patient-related ECHR sites where the patient’s master data are displayed. These could include, for example, name, age, date of birth, residence, diagnoses, allergies, and contact persons.

## 4. Discussion

Given the lack of an ECHR for PPC, the aim of this study, following a DT approach, was to capture the special needs of inpatient and outpatient PPC professionals (physicians, nurses, and secretaries) to be met by an ECHR that supports and facilitates their work. Moreover, an example ECHR based on these needs should be developed as a mock-up.

The participants shed light on the current challenging exchange of information, often characterized by time delays and missing data. From their perspective, an ECHR should quickly share relevant patient information among all the care providers. The participants named medication schedules, an emergency view with the most critical data (e.g., ventilation and medication), an overview of the medical history, the documentation of patient contacts, and an overview of the vital signs relevant to the patient as particularly important criteria for the ECHR. Furthermore, it should be possible to upload documents and exchange information with each other using a patient-specific calendar and a message function. Through this, up-to-date data in emergencies, tracking what other PPC professionals have done, and making a user’s treatments visible to others would be possible. The ECHR should also allow all the providers to have a more comprehensive picture of the most common complex conditions in PPC. A better overview and exchange of the different activities could help to understand correlations that lead to better treatment of symptoms and, thus, increase the quality of care. The ECHR would also make it easier to collect and analyze data to potentially show larger relationships. The ECHR, thus, also holds great potential for research into rare and complex diseases.

The diverse information provided by the users regarding the necessary access authorizations varied widely from patient to patient, entailing that each access authorization should be assigned to individual persons. To keep the effort required to use the ECHR low, the participants wanted to be able to access it from different devices, a simple but also secure login (via password, token, etc.), and link it to existing health record systems. The participants identified a challenge represented by a great variance in the digital competencies of the different users. This may necessitate training for the use of the ECHR since its use by all providers is essential for its success, according to participants. However, it is difficult to develop an ECHR that meets all needs equally. The participants’ opinions were quite diverse in some areas, based on the most common medical conditions of the patients served, the profession, and the place of work. This problem could perhaps be countered by making many aspects of the ECHR customizable to the user (e.g., whether one would like to be notified of new entries by e-mail).

The specific requirements for the ECHR’s design and functionality harbor some points of discussion, which are considered below. One of these was the wish of the participants to grant access to the ECHR to patients and/or their informal caregivers (e.g., parents). Since not all patients in PPC can self-document [5], the involvement of informal caregivers is useful [23]. This certainly offers great opportunities to improve, for example, documentation quality and self-management through a personal health record [24]. Studies on the difficulties of documentation by healthcare professionals suggest that this is a useful approach: Sikroskii et al. revealed a large discrepancy between the severity of oncology patient-reported symptoms and the severity documented by healthcare professionals [25]. Another study identified the advantages of symptom documentation by relatives (e.g., decreased need for communication and more satisfactory symptom management) [26]. In our study, however, the extent of access was discussed, since the ECHR may also contain sensitive content exchanged between PPC professionals that could possibly unsettle parents (e.g., professional discussions). It would certainly be interesting to find out in a further study what the parents themselves think about this possibility and what they require in this respect.

The participants conversed about how much access users should have in the ECHR, not only for parents but also for healthcare professionals. In the FGs, the participants favored a rights system in which users were individually assigned the right to write and read in specific areas (e.g., only medications, not psychological findings). This also carries the risk that users could draw the wrong conclusions from the content because they lack information. In the further course, it is necessary to work out a solution that minimizes this danger, for example by indicating that information is existent but cannot be accessed. The participants would like to have the possibility to limit the access for some users (e.g., for 2 months only). The people who are more involved in care, such as the primary care physician, should have unlimited access. However, the question of which person should determine the respective rights (e.g., patients themselves or the SOPPC team) was unclear and discussed in the FGs.

Closely related to the issue of general access is the need for tracking the changes made by users with access, while security remains one of the greatest challenges in digital health [27]. With regard to the ECHR, it must be taken into account that a large number of users may have access. The participants were concerned that if content can be edited by any user, it will not be possible to trace who made which change. The ECHR could become confusing due to too much data entered in the history of changes. The area of changes in the ECHR is also associated with the need for accountability when posting information.

Some participants expressed a desire to feed entries into the ECHR directly from their own system for electronic health records and vice versa. This interoperability with existing electronic health records is one of the biggest challenges in the adoption [13] and implementation [12] of any electronic health record, especially for ECHRs [28]. To share data, an interface needs to automatically move information from each provider’s existing documentation (e.g., practice information system, PPCU’s electronic health record) into the ECHR and to transfer information from the ECHR into their own documentation. The information that must be transferred between the systems needs to be correctly semantically encoded to be useful [29]. Such semantic code already exists for many of the desired contents, since they are common parameters (e.g., medication schedule, blood pressure, etc.). However, other requested content is so specific to the PPC (e.g., comprehensive symptom documentation) that the correct semantic encoding of those items is needed first. Such challenges have a major impact on the possible implementation and resulting acceptance of the ECHR and must, therefore, be considered as priorities in the following.

In summary, the needs assessment showed that there are very specific requirements in PPC. There are already existing ECHRs for other settings in Germany. Yet, their functionality only includes sending electronic documents between facilities (e.g., the PDF of a medication plan) [30]. The participants made it clear that content should be editable collaboratively and that communication should also be possible using the ECHR (e.g., via messaging function). Such functions are not available in the existing systems in Germany. This illustrates the necessity of the approach used here for new development with users in order to take needs into account and, thus, improve the quality of care with the introduction of an ECHR.

Following the substantive and functional discussion points regarding the ECHR, the following section discusses the usefulness of DT for achieving research objectives. As noted in a review of DT in agile software development, DT helped to facilitate communication between the participants and the researchers and may lead to an increase in a software’s quality and user satisfaction [31]. Additionally, in this study, working with mock-ups ensured a specific exchange with PPC professionals about their ECHR needs. This facilitated a detailed presentation of content based on user needs and the clarification of potential misunderstandings. It also offered a better, comprehensive overview of the content and functionality that users wanted. This allowed potential linkages to be identified together with an overabundance of information in one view during an early stage of the ECHR’s development.

Significantly, the diversity of the participants resulted in a wide range of views and needs. The challenge, then, was to reconcile these disparate needs, some of which were sector-specific. The DT model of, first, conducting the occupational group and setting-specific interviews for the initial needs’ assessment, followed by the joint discussion and exploration of the needs in the cross-occupational group and cross-setting FGs proved helpful in this regard.

Another challenge for the study was the abstract nature of the interview conversations, in contrast to the complex subject matter of the ECHR. This required a great deal of creativity and understanding of the setting and needs of the participants. Here, the DT model of iteration allowed for constant reassurance and specification. Another challenging aspect was some participants’ limited experience with electronic documentation. The DT elements were helpful once more—the mock-ups in particular vividly illustrated the team’s ideas, on the basis of which joint discussions were held. Similar challenges emerged in Nguyen et al.’s study of palliative care professionals’ assessments of technology use [32]. In Nguyen et al.’s study, the participants’ level of experience and/or knowledge regarding technologies also varied. This resulted in some participants’ limited ability to provide feedback on certain aspects. Nevertheless, the study described the inclusion of all the users as a valuable reflection of the breadth of potential users [32]. This can also be applied to our study, where users with different experiences shaped the diversity of ideas.

It was challenging to transform participants’ wishes, which were often formulated globally, into specific needs. For this purpose, the application of user stories was helpful, since they identified which aim triggered a need (“*I need this in order to...*”) [33]. The formulation in user stories could, thus, clarify when the reason for a need was not known. In this case, it could be asked again in the following survey steps. The use of user stories has already been positively evaluated in a study on the development of a part of an electronic health record involving healthcare professionals. The authors described the listing of “who”, “what”, and “why” in the user stories as helpful in understanding needs. However, just as in our study, the authors also mentioned that the “why” of the user stories was not often clear and had to be continuously asked and expanded upon. In the process, a continuous review of the created user stories was crucial to ensure that the final product met their needs [34]. This is where the iterative DT model proved helpful in our study.

## 5. Limitations

This study is a first step towards the development of an ECHR for PPC in Germany. The study was conducted exclusively in Germany with a small sample of *n*= 36 individuals. In addition, only personnel from the PPCU, SOPPC team, and medical offices were interviewed. There are many other individuals (e.g., psychosocial staff) involved in the holistic care of patients in the PPC who may also need access to the ECHR. These individuals make an important contribution to complementing the holistic approach to treatment, including the ECHR. Significantly, general practitioners were not interviewed regarding the specific number of PPC patients cared for and their conditions. Due to the wide variance in symptoms and needs of PPC patients, this may impact the needs named by participants regarding an ECHR. Therefore, the results should not be considered representative across the different settings of PPC. Further research in different settings (e.g., teams that primarily care for oncology patients or teams that primarily care for patients with rare genetic diseases) is needed to refine the needs for an ECHR in PPC. Nevertheless, this study should be viewed as a prelude to further research into the enormously important digital networking of professionals in PPC.

## 6. Conclusions

Using the DT model as a theoretical framework, the needs of PPC professionals regarding an ECHR were obtained and an example ECHR was conceptualized.

The future development and implementation of an ECHR in the PPC setting could help ensure that critical information (e.g., allergies, medications, advance care plan) is available and up to date in an emergency, save time in sharing information, and provide all the stakeholders with a more comprehensive view of the past and current medical history of patients. This can also help prepare future treatment wisely and prevent redundancy. These changes would have a positive effect on patients, the safety and quality of their care, and the resulting outcomes.

Finally, in the future, it will be necessary to clarify the preferences of the patients and their relatives in terms of access rights and whether they desire the ability to make entries in the ECHR themselves.

## Figures and Tables

**Table 1:** Adapted DT model as the methodological basis of this study [15].

Step	Aim of the DT-Step	Study Methodology
Needs Assessment		
1. Empathy ^1^	This step is characterized by understanding how PPC professionals exchange information about patients. The focus here is the needs of PPC professionals in terms of sending and receiving information.	➔ Interviews Inquiring about the current situation and the needs for intersectoral information exchange
2. Define	In this step, it is crucial to capture how the ECHR should be designed, who the development is relevant to, which framework conditions influence the ECHR, and which target state should be achieved from the users’ perspective.	➔ Analysis + User Stories Investigate who is sharing case-related information, when, through what media, and what users desire from an ECHR. The analysis was oriented to the UTAUT. The resulting needs were formulated as user stories (e.g., “As a physician, I would like to have an overview of the current medication in order to have a direct overview in case of an emergency admission to the ward”).
3. Ideate	This step focuses on idea generation to produce innovative solutions for PPC professionals.	➔ Brainstorming Group discussion regarding the ECHR’s technical implementation and content design.
Conceptualization		
4. Prototype	Prototyping helps generate a demonstrative solution that can appeal to PPC professionals without heavily investing their money or time. Discussions and ideas are substantiated, and misunderstandings can be reduced.	➔ Designing Prototypes Preparation of mock-ups based on the user stories.
5. Test ^1^	This step serves the following purposes: (a) inspiration for the research and development team, (b) inspiration for the users, and (c) evaluation of the criteria that were considered to develop the prototype.	➔ Focus Group Discussions (FGs) Presentation of the mock-ups during discussions with professionals, guided by specific questions. In the FGs, personas, a patient journey map, and a stakeholder map were used to encourage creativity among participants.
6. Iteration	Here, steps 1–4 were repeated to reiterate the already specified needs as a basis for software programming.	➔ Adaption of the Prototypes FGs were analyzed and the needs verbalized as user stories to understand and define additional user needs. The existing prototypes were adapted by the research and development team after they brainstormed possible solutions.

^1^ Steps requiring the active inclusion and interviewing of PPC professionals.

**Table 2:** Characteristics of participants.

	PPCU ^a^	SOPPC ^b^	PPCU/SOPPC ^c^	MO ^d^
Sex (*n*)				
Female	5	1	2	5
Male	2	3	1	8
Age in years (*M*)	47.6	41.0	53.0	52.8
Profession (*n*)				
Nurse	1	2	1	-
Physician	4	2	2	13
Secretary	2	-	-	-
Years of work experience (*n*)				
0–9	2	1	-	1
10–19	-	2	1	3
20–29	2	1	-	4
≥30	3	-	2	5
Years of experience in current position (*n*)				
0–4	3	1	2	2
5–8	1	2	-	2
9–11	1	1	1	-
≥12	2	-	-	9
Experience in electronic documentation (*n*)				
	2	2	2	5
Experience in electronic documentation in years (*n*)				
0–4	1	1	-	2
5–8	-	1	-	-
9–11	1	-	1	-
≥12	-	-	1	3

^a^ *n* = 7; ^b^ *n* = 7 (*n* = 1 physician and *n* = 2 nurses characteristics are missing); ^c^ *n* = 4 (*n* = 1 physician characteristic is missing); ^d^ *n* = 18 (*n* = 5 physicians characteristics are missing); Missing data resulted from a failure to return context data by the participants.

## Data Availability

The corresponding datasets of this study are available from the corresponding author upon reasonable request.

## Supplementary Materials

The following are available online at https://www.mdpi.com/article/10.3390/children8070602/s1, Figure S1: Patient journey map. Figure S2: Stakeholder map. Figure S3: Graphic Recording.
