# Supplementary material for: Needs Assessment for the Development of an Electronic Cross-Facility Health Record (ECHR) for Pediatric Palliative Care: A Design Thinking Approach
Source: Children (Basel). 2021 Jul 16;8(7):602. doi: 10.3390/children8070602 (PMC8304612; doi:10.3390/children8070602)
Supplement: Supplementary file 1 [file children-08-00602-s001.zip › Supplementary Files Busse et al Revised/Appendix 3 - Graphic Recording.pdf]

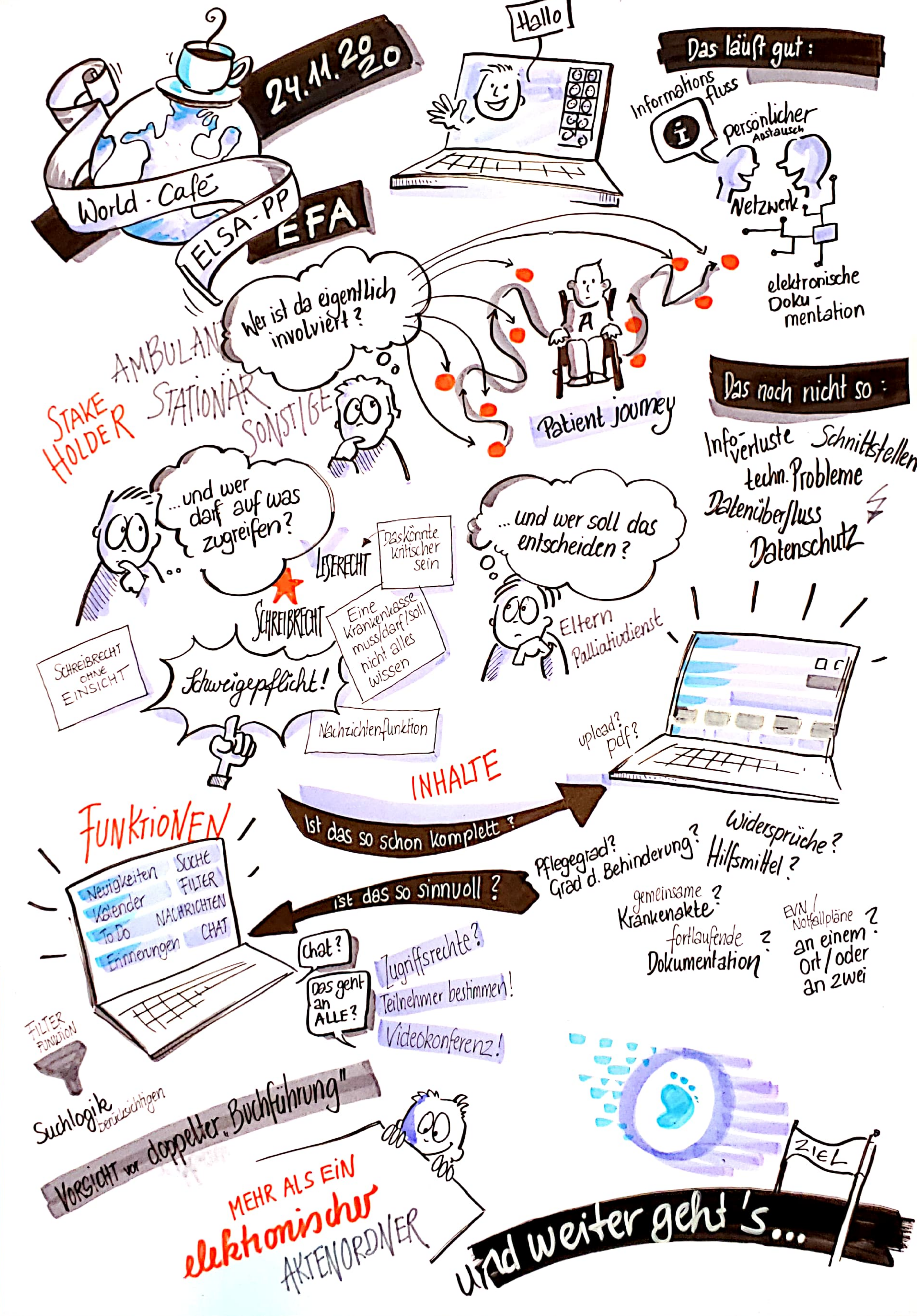

World-Café
24.11.2020
ELSA-PP
EFA
Hallo
Das läuft gut:
Informationsfluss
persönlicher Austausch
Netzwerk
elektronische Doku-mentation
Wer ist da eigentlich involviert?
Patient journey
A
STAKEHOLDER
AMBULANT
STATIONÄR
SONSTIGE
Das noch nicht so:
Info-verluste
Schnittstellen
techn. Probleme
Datenüberfluss
Datenschutz
...und wer darf auf was zugreifen?
LESERECHT
Das könnte kritischer sein
SCHREIBRECHT
Eine Krankenkasse muss/darf/soll nicht alles wissen
SCHREIBRECHT OHNE EINSICHT
Schweigepflicht!
Nachrichtenfunktion
...und wer soll das entscheiden?
Eltern
Palliativdienst
upload? pdf?
INHALTE
Ist das so schon komplett?
FUNKTIONEN
Neuigkeiten
Kalender
To Do
Erinnerungen
SUCHE
FILTER
NACHRICHTEN
CHAT
Ist das so sinnvoll?
Pflegegrad?
Grad d. Behinderung?
Widersprüche?
Hilfsmittel?
gemeinsame Krankenakte?
fortlaufende Dokumentation?
EVN / Notfallpläne?
an einem Ort / oder an zwei
Chat?
Das geht an ALLE?
Zugriffsrechte?
Teilnehmer bestimmen!
Videokonferenz!
FILTER FUNKTION
Suchlogik berücksichtigen
VORSICHT vor doppelter „Buchführung"
MEHR ALS EIN elektronischer AKTENORDNER
ZIEL
und weiter geht's...

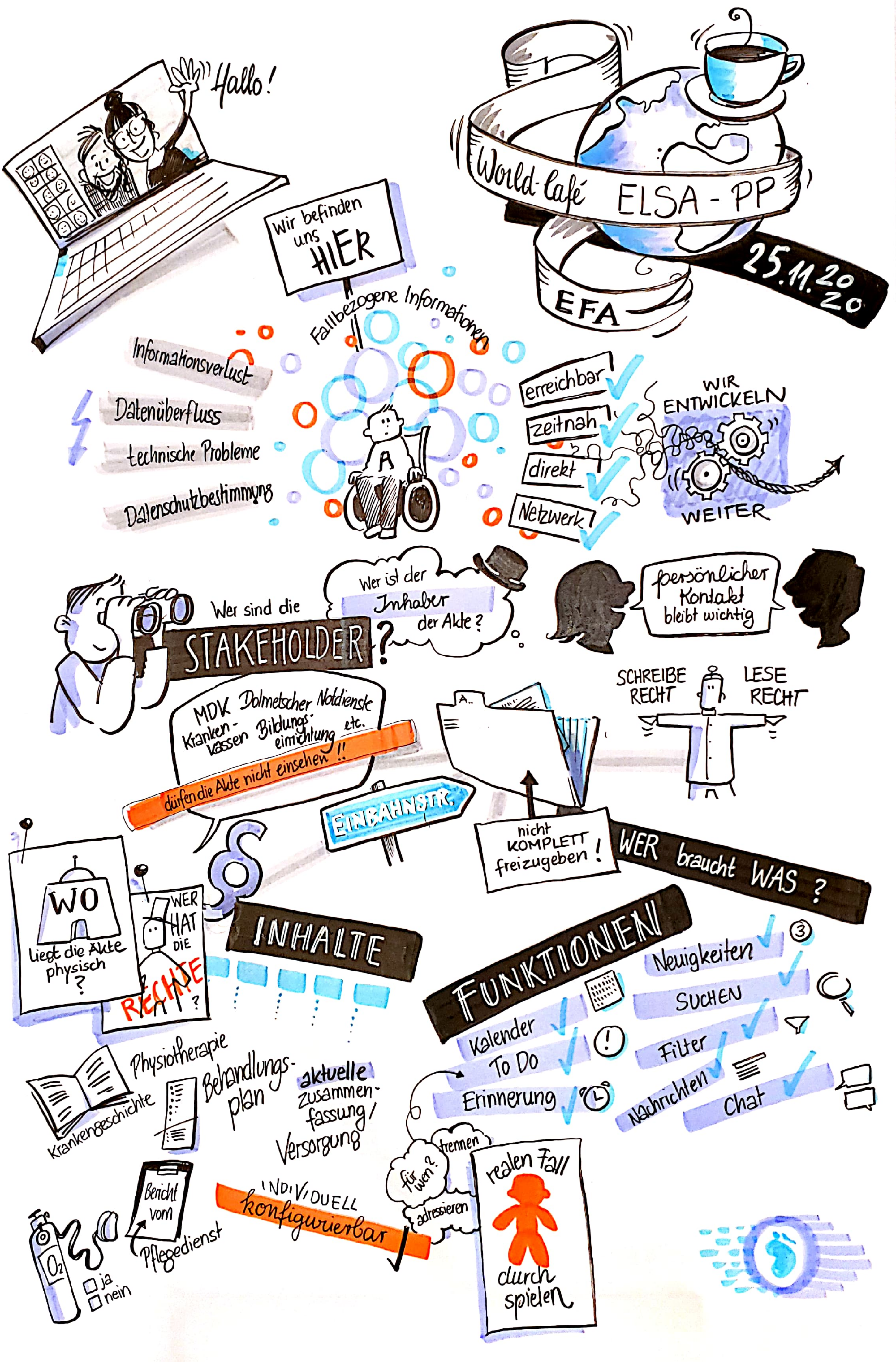

Hallo!
World-Café ELSA-PP
EFA
25.11.2020
Wir befinden uns HIER
Fallbezogene Informationen
Informationsverlust
Datenüberfluss
technische Probleme
Datenschutzbestimmung
A
erreichbar
zeitnah
direkt
Netzwerk
WIR ENTWICKELN WEITER
Wer ist der Inhaber der Akte?
Wer sind die STAKEHOLDER?
persönlicher Kontakt bleibt wichtig
SCHREIBE RECHT
LESE RECHT
MDK Dolmetscher Notdienste Kranken-kassen Bildungs-einrichtung etc.
dürfen die Akte nicht einsehen!!
EINBAHNSTR.
nicht KOMPLETT freizugeben!
WER braucht WAS?
WO liegt die Akte physisch?
WER HAT DIE RECHTE?
INHALTE
FUNKTIONEN
Neuigkeiten
SUCHEN
Kalender
To Do
Erinnerung
Filter
Nachrichten
Chat
Krankengeschichte
Physiotherapie
Behandlungs-plan
aktuelle Zusammen-fassung/ Versorgung
Bericht vom Pflegedienst
O_2
ja
nein
INDIVIDUELL konfigurierbar
für wen?
trennen
adressieren
realen Fall durchspielen

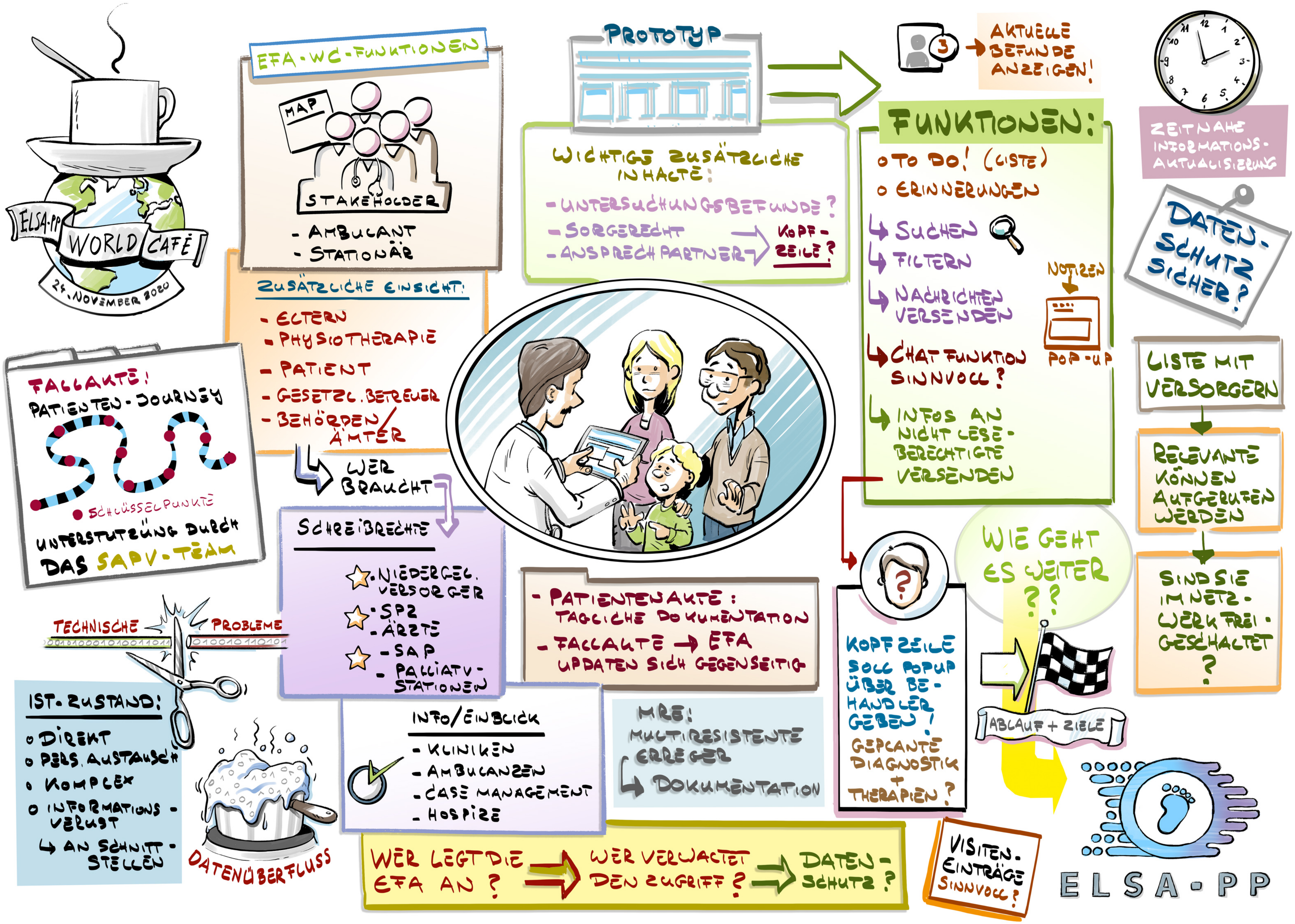
ELSA-PP
WORLD CAFÉ
24. NOVEMBER 2020
EFA-WC-FUNKTIONEN
MAP
STAKEHOLDER
- AMBULANT
- STATIONÄR
ZUSÄTZLICHE EINSICHT:
- ELTERN
- PHYSIOTHERAPIE
- PATIENT
- GESETZL. BETREUER
- BEHÖRDEN/ÄMTER
WER BRAUCHT?
FALLAKTE!
PATIENTEN-JOURNEY
SCHLÜSSELPUNKTE
UNTERSTÜTZUNG DURCH DAS SAPV-TEAM
SCHREIBRECHTE
- NIEDERGEL. VERSORGER
- SPZ
- ÄRZTE
- SAP
- PALLIATIV-STATIONEN
TECHNISCHE PROBLEME
IST-ZUSTAND:
o DIREKT
o PERS. AUSTAUSCH
o KOMPLEX
o INFORMATIONS-VERLUST
↳ AN SCHNITT-STELLEN
DATENÜBERFLUSS
INFO/EINBLICK
- KLINIKEN
- AMBULANZEN
- CASE MANAGEMENT
- HOSPIZE
PROTOTYP
WICHTIGE ZUSÄTZLICHE INHALTE:
- UNTERSUCHUNGSBEFUNDE?
- SORGERECHT
- ANSPRECHPARTNER
KOPF-ZEILE?
- PATIENTENAKTE: TÄGLICHE DOKUMENTATION
- FALLAKTE → EFA UPDATEN SICH GEGENSEITIG
MRE: MULTIRESISTENTE ERREGER
↳ DOKUMENTATION
WER LEGT DIE EFA AN? → WER VERWALTET DEN ZUGRIFF? → DATEN-SCHUTZ?
AKTUELLE BEFUNDE ANZEIGEN!
FUNKTIONEN:
o TO DO! (LISTE)
o ERINNERUNGEN
↳ SUCHEN
↳ FILTERN
↳ NACHRICHTEN VERSENDEN
↳ CHAT FUNKTION SINNVOLL?
NOTIZEN
POP-UP
↳ INFOS AN NICHT LESE-BERECHTIGTE VERSENDEN
ZEITNAHE INFORMATIONS-AKTUALISIERUNG
DATEN-SCHUTZ SICHER?
LISTE MIT VERSORGERN
RELEVANTE KÖNNEN AUFGERUFEN WERDEN
SIND SIE IM NETZ-WERK FREI-GESCHALTET?
WIE GEHT ES WEITER??
KOPFZEILE SOLL POPUP ÜBER BE-HANDLER GEBEN!
GEPLANTE DIAGNOSTIK + THERAPIEN?
ABLAUF + ZIELE
VISITEN-EINTRÄGE SINNVOLL?
ELSA-PP

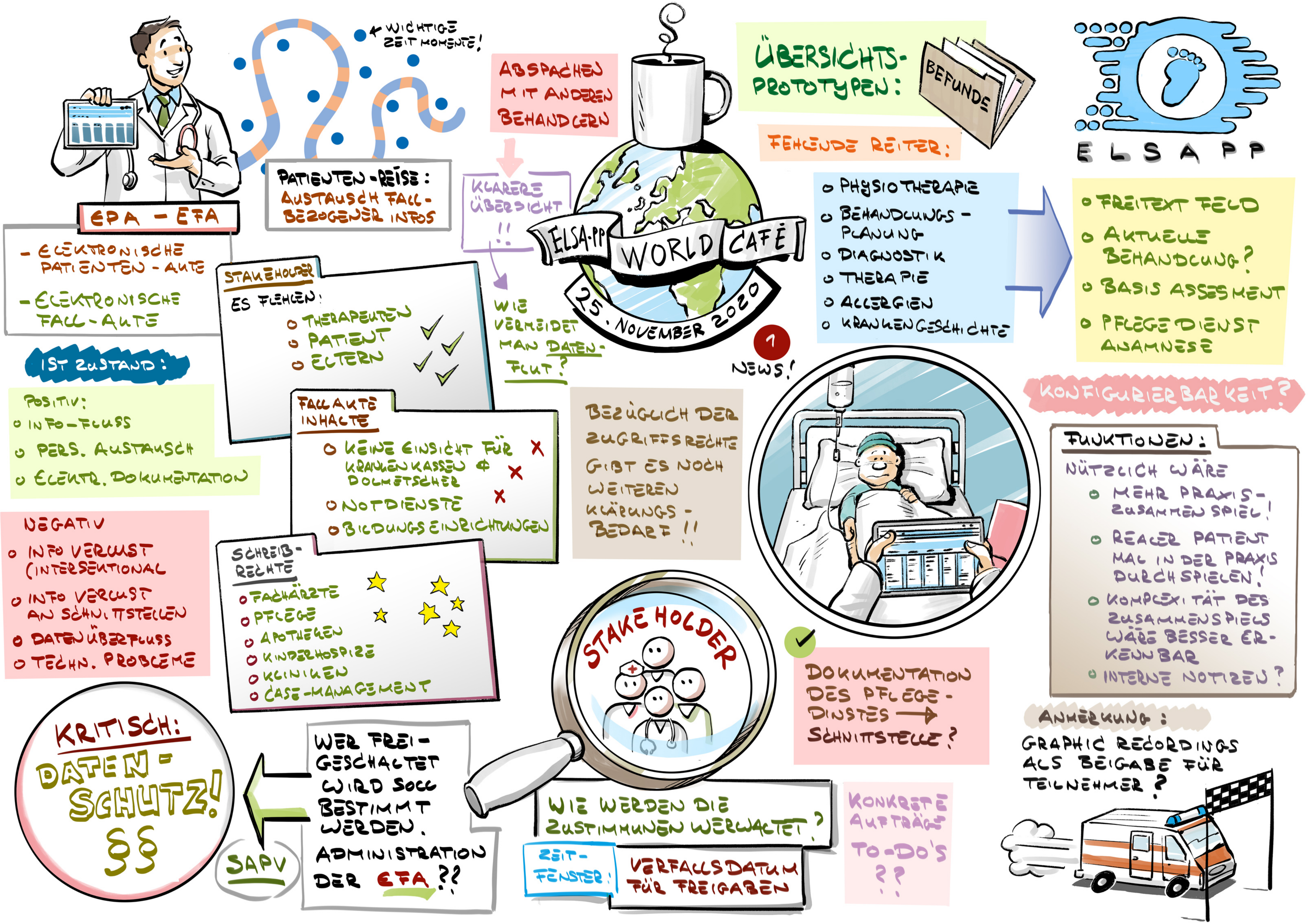

WICHTIGE ZEITMOMENTE!
PATIENTEN-REISE: AUSTAUSCH FALL-BEZOGENER INFOS
ABSPRACHEN MIT ANDEREN BEHANDLERN
KLARERE ÜBERSICHT !!
WIE VERMEIDET MAN DATEN-FLUT?
ELSA-PP WORLD CAFÉ
25. NOVEMBER 2020
ÜBERSICHTS-PROTOTYPEN:
BEFUNDE
FEHLENDE REITER:
o PHYSIOTHERAPIE
o BEHANDLUNGS-PLANUNG
o DIAGNOSTIK
o THERAPIE
o ALLERGIEN
o KRANKENGESCHICHTE
ELSAPP
o FREITEXT FELD
o AKTUELLE BEHANDLUNG?
o BASIS ASSESMENT
o PFLEGEDIENST ANAMNESE
EPA - EFA
- ELEKTRONISCHE PATIENTEN-AKTE
- ELEKTRONISCHE FALL-AKTE
IST ZUSTAND:
POSITIV:
o INFO-FLUSS
o PERS. AUSTAUSCH
o ELEKTR. DOKUMENTATION
NEGATIV
o INFO VERLUST (INTERSEKTIONAL
o INFO VERLUST AN SCHNITTSTELLEN
o DATENÜBERFLUSS
o TECHN. PROBLEME
STAKEHOLDER
ES FEHLEN:
o THERAPEUTEN
o PATIENT
o ELTERN
FALLAKTE INHALTE
o KEINE EINSICHT FÜR KRANKENKASSEN & DOLMETSCHER
o NOTDIENSTE
o BILDUNGSEINRICHTUNGEN
SCHREIB-RECHTE
o FACHÄRZTE
o PFLEGE
o APOTHEKEN
o KINDERHOSPIZE
o KLINIKEN
o CASE-MANAGEMENT
BEZÜGLICH DER ZUGRIFFSRECHTE GIBT ES NOCH WEITEREN KLÄRUNGS-BEDARF !!
1 NEWS!
KONFIGURIERBARKEIT?
FUNKTIONEN:
NÜTZLICH WÄRE
o MEHR PRAXIS-ZUSAMMENSPIEL!
o REALER PATIENT MAL IN DER PRAXIS DURCHSPIELEN!
o KOMPLEXITÄT DES ZUSAMMENSPIELS WÄRE BESSER ERKENNBAR
o INTERNE NOTIZEN?
STAKEHOLDER
DOKUMENTATION DES PFLEGE-DINSTES → SCHNITTSTELLE?
ANMERKUNG:
GRAPHIC RECORDINGS ALS BEIGABE FÜR TEILNEHMER?
KRITISCH: DATEN-SCHUTZ! §§
SAPV
WER FREIGESCHALTET WIRD SOLL BESTIMMT WERDEN.
ADMINISTRATION DER EFA??
WIE WERDEN DIE ZUSTIMMUNGEN VERWALTET?
ZEIT-FENSTER!
VERFALLSDATUM FÜR FREIGABEN
KONKRETE AUFTRÄGE
TO-DO'S ??
