# Supplementary figures and images for: Needs Assessment for the Development of an Electronic Cross-Facility Health Record (ECHR) for Pediatric Palliative Care: A Design Thinking Approach
Source: Children (Basel). 2021 Jul 16;8(7):602. doi: 10.3390/children8070602 (PMC8304612; doi:10.3390/children8070602)

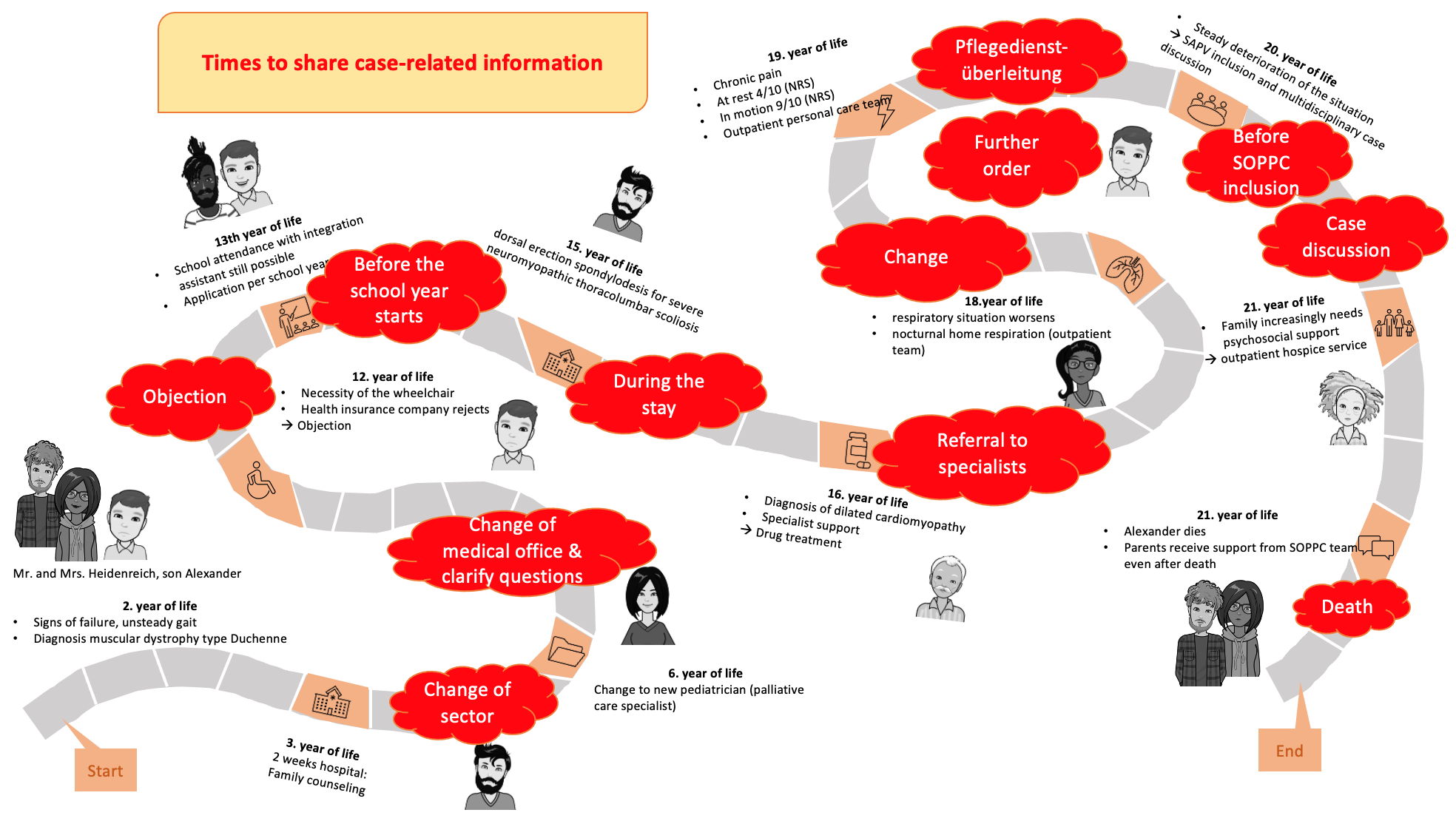

Supplement: Supplementary file 1 [file children-08-00602-s001.zip › Supplementary Files Busse et al Revised/Appendix 1 - Patient Journey Map.png]

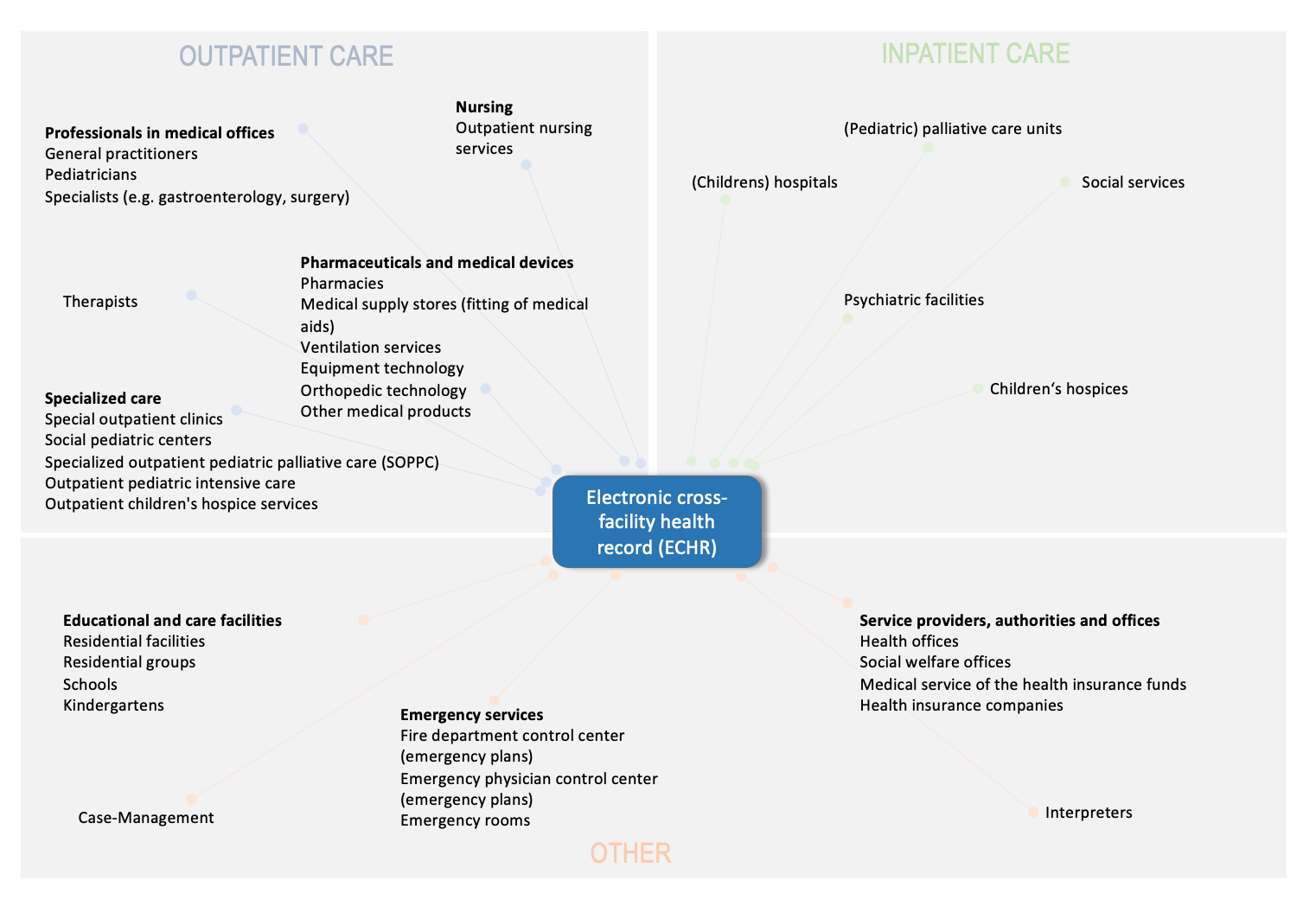

Supplement: Supplementary file 1 [file children-08-00602-s001.zip › Supplementary Files Busse et al Revised/Appendix 2 - Stakeholder map.png]
